# Intracoronary Imaging for Changing Therapeutic Decisions in Patients with Multivascular Coronary Artery Disease

**DOI:** 10.3390/medicina60081320

**Published:** 2024-08-15

**Authors:** Dan Pasaroiu, Imre Benedek, Teodora Popa, Constantin Tolescu, Monica Chitu, Theodora Benedek

**Affiliations:** 1 Clinic of Cardiology, Mures, County Emergency Clinical Hospital, 540142 Târgu Mures, Romania; dan.pasaroiu@yahoo.com (D.P.); imre.benedek@umfst.ro (I.B.); cristi.tolescu95@gmail.com (C.T.); iuliachitu@yahoo.com (M.C.); theodora.benedek@umfst.ro (T.B.); 2 Center of Advanced Research in Multimodality Cardiac Imaging, CardioMed Medical Center, 540124 Târgu Mures, Romania; 3 Doctoral School of Medicine and Pharmacy, University of Medicine, Pharmacy, Science and Technology “George Emil Palade” of Târgu Mures, 540139 Târgu Mures, Romania

**Keywords:** vulnerable plaque, non-culprit lesion, optical coherence tomography, fractional flow reserve

## Abstract

*Background and Objectives*: Atherosclerotic disease is a major contributor to heart failure, stroke, and myocardial infarction, significantly lowering the quality of life and life expectancy and placing a significant burden on healthcare. Not all lesions deemed non-significant are benign, and conversely, not all significant lesions are causative of ischemia. Fractional flow reserve (FFR) provides a functional assessment of coronary lesions, while optical coherence tomography (OCT) offers detailed imaging of plaque morphology, aiding in therapeutic decision-making. The objective of this study was to evaluate the utility of OCT and FFR as adjunctive tools in the catheterization laboratory for guiding therapeutic decisions in patients with multivessel disease for non-culprit vessels. Specifically, we aimed to assess how OCT and FFR influence therapeutic decision-making in patients with multivessel coronary artery disease. *Materials and Methods*: A total of 36 patients with acute coronary syndrome (ACS) and multivessel disease were randomized 1:1 into two groups: one guided by FFR alone and the other by a combination of FFR and OCT. For the FFR group, revascularization decisions for non-culprit lesions were based solely on FFR measurements. If the FFR was >0.8, the procedure was concluded, and the patient received maximal medical treatment. If the FFR was ≤0.8, a stent was placed. For the FFR + OCT group, if the FFR was >0.8, the revascularization decision was based on OCT findings. If there were no vulnerable plaques (VP), the procedure was concluded, and the patient received maximal medical treatment. If OCT imaging indicated VP, then the patient underwent revascularization. If the FFR was ≤0.8, the patient underwent revascularization regardless of OCT findings. *Results*: OCT imaging altered the therapeutic decision in 11 cases where FFR measurements were above 0.8, but the lesions were characterized as VP. Analyzing the total change in the decision to stent, 4 cases in the FFR group and 15 cases in the FFR and OCT groups (4 based on FFR and 11 on OCT) revealed a statistically significant difference (*p* = 0.0006; Relative Risk = 0.2556; 95% CI: 0.1013 to 0.5603). When analyzing the change in the total decision both to stent and not to stent, we observed a statistically significant difference, with Group 1 having 7 cases and Group 2 having 15 cases (*p* = 0.0153; Relative Risk = 0.4050; 95% CI: 0.2004 to 0.7698. *Conclusions*: Based on the findings of this study, OCT significantly increases the percentage of stenting procedures by identifying vulnerable lesions. The use of intracoronary imaging facilitates the timely identification and treatment of these vulnerable lesions. This underscores the crucial role of OCT in enhancing the precision of coronary interventions by ensuring timely intervention for vulnerable lesions, thereby potentially improving patient outcomes.

## 1. Introduction

The most common and dangerous illness in the world, atherosclerotic disease, greatly increases the morbidity and death rate worldwide, with 3.9 million people dying every year from its consequences. The accumulation of cholesterol and inflammatory cells on the artery walls causes atherosclerosis, which leads to the narrowing of the arterial vessels, increased stiffness of their walls, and decreased blood flow in the atherosclerotic arteries. Although the death rate started to decline in recent years in European countries, including those in Central and Eastern Europe, the incidence of atherosclerotic disease is rising, particularly in middle- and low-income countries, where urbanization and lifestyle changes led to the adoption of unhealthy dietary and behavioral habits. Atherosclerotic disease is a major contributor to heart failure, stroke, and myocardial infarction, significantly lowering the quality of life, life expectancy and placing a significant burden on healthcare. Globally, controlling and preventing atherosclerotic disease is a top priority for public health.

Diagnosis of significant coronary artery stenosis requires contrast opacification of the coronary vessels using a contrast material that is injected into the coronary ostium during invasive angiography. An alternative technique for visualization of coronary arteries is non-invasive CT angiography, which uses the intravenous injection of a contrast agent followed by a CT scan. However, AngioCT remains a diagnostic test [1]. When AngioCT identifies a significant stenosis, the case is referred to the cath lab for percutaneous revascularization and stent implantation.

It is important to remember that not all flow-limiting stenoses produce ischemia. Fractional flow reserve (FFR) is a technique frequently used in cardiac catheterization laboratories for the determination of the functional significance of a coronary lesion. It calculates the ratio between the maximum theoretical flow in a healthy coronary artery and the maximum achievable blood flow in an afflicted coronary artery. An FFR higher than 0.8 is regarded as normal; however, an FFR of less than 0.75 to 0.80 is typically linked to myocardial ischemia (MI) [2]. Using a pressure wire to assess the aortic pressure in the event of maximal myocardial hyperemia and the distal coronary pressure at stenosis, this approach can be carried out during coronary angiography [2]. Since FFR measurement offers an unbiased, quantitative evaluation of the functional severity of coronary stenoses, its application in cardiac catheterization laboratories has grown in frequency.

At the same time, not all the non-significant lesions are innocent. Some of them may carry inside them the so-called markers of vulnerability that may expose them to an unpredictable evolution towards accelerated progression, rupture, and the development of acute coronary syndrome (ACS). The capacity of visual angiography to differentiate between stable lesions and those that potentially cause MI is limited since these are not directly related to the degree of vessel narrowing [3]. Therefore, additional imaging techniques like intravascular ultrasound (IVUS) or optical coherence tomography (OCT) are often utilized to provide more detailed information about plaque morphology and composition, helping to guide optimal treatment decisions [4].

A coronary atheromatous plaque becomes unstable when it has a large necrotic lipid core and a thin fibrous cap infiltrated by macrophages. This instability is often accompanied by active vascular remodeling, where the vessel diameter enlarges at the plaque site and spotty calcifications are present within the plaque [5]. The most commonly used intracoronary imaging methods for the assessment of plaque vulnerability are intravascular ultrasound (IVUS) and optical coherence tomography. Due to its high resolution (10–15 µm), OCT can distinguish between the internal and external elastic laminae, as well as differentiate between the intima/media/adventitia layers, which is not possible with the lower resolution (100–150 µm) of intravascular ultrasound [3]. Thin-cap fibroatheroma (TCFA) are plaques that are susceptible to rupture and have thin fibrous caps with a cutoff of 70 µm, rich in macrophages overlying a large lipid-rich necrotic core. Ruptured plaques refer to areas within an arterial plaque that have developed a tear or rupture. This rupture allows the contents of the plaque, such as lipids and cellular debris, to encounter the bloodstream, which leads to the formation of a thrombus. Unlike plaque rupture, plaque erosion is characterized by the presence of a luminal thrombus and the absence of the endothelium, with no evidence of disruption of the fibrous cap. Thanks to the high resolution of OCT, it is well-suited for identifying these high-risk plaques [3].

The aim of the present study was to assess the role of OCT and FFR as adjunctive tools in the cathlab for establishing the therapeutic decision in patients with multivascular disease in whom the results of angiography are inconclusive for the non-culprit vessel. Specifically, we aimed to investigate the impact of OCT and FFR on changing the therapeutic strategy in patients with bi- or three-vessel coronary artery disease (CAD).

## 2. Materials and Methods

This is a single-center prospective randomized study investigating the role of FFR and OCT imaging in changing the therapeutic decision for non-culprit lesions in patients with acute coronary syndromes and multivessel CAD.

### 2.1. Study Population

Thirty-six patients with acute coronary syndrome defined as unstable angina (UA), non-ST-segment elevation myocardial infarction (NSTEMI), and ST-segment elevation myocardial infarction (STEMI) who underwent percutaneous coronary intervention (PCI) at the Cardiology Clinic of the Târgu Mureș County Emergency Clinical Hospital between January 2022 and May 2024 were enrolled. The inclusion criteria comprised patients under 80 years of age presenting with acute coronary syndrome and multivessel disease (MVD), defined as one or more vessels affected by at least 60% stenosis, who demonstrated willingness to participate in the study. Exclusion criteria included patients with MVD necessitating coronary artery bypass grafting (CABG), those over 80 years of age, lesions situated in a grafted segment or vein graft, or instances where FFR and OCT were considered infeasible or hazardous due to patient anatomy. The patients were randomly assigned to either the FFR-guided (Group 1) revascularization or the FFR&OCT-guided (Group 2) revascularization. Lesions responsible for ACS underwent stenting in accordance with current guidelines. We evaluated the non-culprit lesions (not responsible for ACS) either using FFR alone for Group 1 or a combination of FFR and OCT for Group 2. In total, 18 patients were assigned to each group.

A UA/NSTEMI/STEMI was defined according to the standard criteria: STEMI was defined as a new ST-segment elevation of ≥1 mm at the J-point in two or more contiguous leads accompanied by an elevation of troponin levels above the 99th percentile. NSTEMI was defined as a new ST-segment depression > 0.1 mm or T wave inversion of at least 0.3 mm in more than 2 contiguous leads accompanied by an elevation of troponin levels above the 99th percentile, and a UA was defined as a Canadian Cardiology Society ischemic discomfort class 3 or 4 accompanied by ST/T changes on electrocardiogram (ECG) but without ST-segment elevation and without an increase in troponin levels.

### 2.2. Methodology

All patients were screened for the presence of risk factors (diabetes, smoking, dyslipidemia, hypertension, family history of heart disease, history of ACS, and prior PCI). Diabetes mellitus was defined as patients having fasting plasma glucose ≥ 126 mg/dL and/or post-prandial plasma glucose ≥ 200 mg/dL and/or A1c ≥ 6.5% or a history of diabetes and/or taking medication for diabetes. Hypertension was defined as systolic blood pressure ≥ 140 mmHg and/or diastolic blood pressure ≥90 mmHg and/or antihypertensive treatment. Dyslipidemia was defined as an LDL > 100 mg/dL and/or cholesterol > 200 mg/dL and/or triglycerides > 200 mg/dL and/or on hypolipidemic treatment. A history of cardiovascular disease was defined as having a first-degree relative with atherosclerotic cardiovascular disease at an age younger than 55 years. Smoking was defined as either current or before hospitalization.

### 2.3. Procedures

All patients underwent PCI of the culprit lesion responsible for ACS; FFR measurements and OCT imaging for non-culprit lesions were performed during the PCI procedure or during a staged procedure. 

### 2.4. Urgent Coronary Angiography

Patients presenting with ACS were referred to our Cath Lab, where a 6 French sheath was inserted into either the radial, brachial, or femoral artery, and 7500 IU of heparin was administered before starting the procedure. Angiography imaging was performed using the Philips Azurion 7 B20 system (Veenpluis, The Netherlands). All lesions deemed responsible for ACS were revascularized according to current guidelines. If one or more vessels had at least 60% stenosis and were suitable for FFR and OCT evaluation, the study terms were explained to the patient. After obtaining written consent, FFR measurements and OCT imaging were performed either in the same session or as a staged procedure. The decision for a staged procedure was based on either patient preference or the interventional cardiologist’s recommendation. If a staged procedure was performed at a later date, the sheath was removed, hemostasis was achieved, and a new sheath was inserted at the start of the staged procedure, with 7500 IU of heparin administered.

### 2.5. FFR Measurements

**Figure 1:**
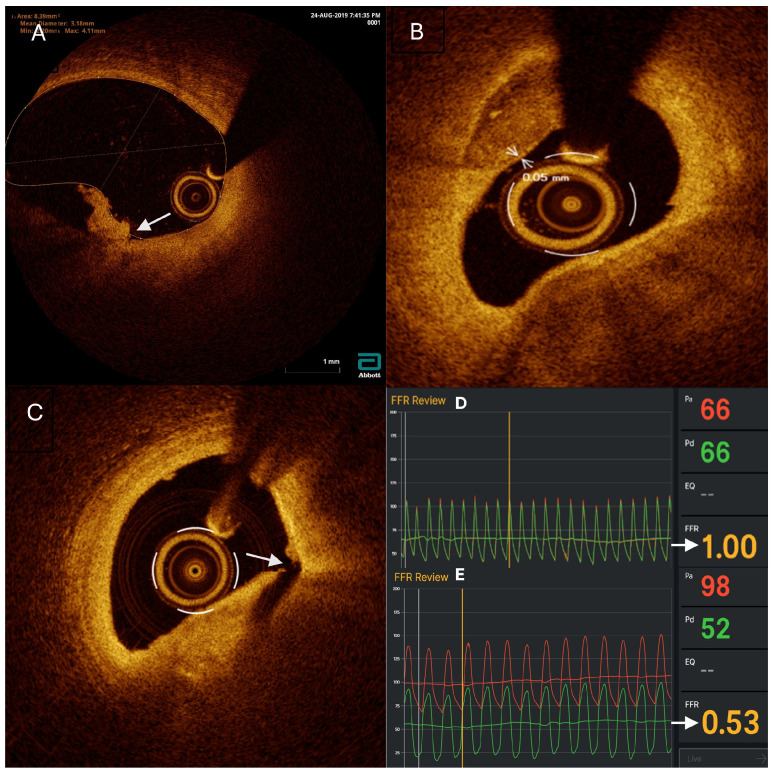
Intracoronary assessment, imaging, and physiology for modifying therapeutic decisions. (**A**) Example of OCT image with plaque erosion with intraluminal thrombus (white arrow). (**B**) Example of an OCT image with plaque with a 0.05 mm fibrous cap thickness (white arrows). (**C**) Example of an OCT image with plaque rupture (white arrow). FFR recordings. (**D**) Example of negative FFR with a value of 1 (white arrow) with no indication for revascularization. (**E**) Example of a positive FFR with a value of 0.53 (white arrow) with an indication for revascularization.

FFR measurements of the non-culprit lesions were performed in both groups using the Abbott PressureWire X 0.014 (0.36 mm) × 175 cm (Coyol Alajuela, Costa Rica), positioned at the target lesion. FFR at baseline, as well as the ratio of the pressure distal (PD) to coronary stenosis to the aortic pressure (PA) (Pd/Pa) and the Resting Full-cycle Ratio (RFR), were recorded. After baseline recordings, adenosine was injected into the coronary artery (200 micrograms for the left coronary artery and 100 micrograms for the right coronary artery), and a second FFR measurement was obtained for each non-culprit lesion. Figure 1 exemplifies FFR measurements with and without indication for revascularization.

### 2.6. OCT Imaging

OCT imaging was performed only in the FFR and OCT groups using the Abbott Dragonfly OpStar Imaging Catheter (1.78 mm/0.36 mm/135 cm) (Westford, MA, USA). Upon reaching the target lesion, the catheter was flushed with a saline solution, followed by an injection of contrast media, and an OCT pullback was performed. The images were recorded using Abbott’s OPTIS™ Integrated Next (Westford, MA, USA) and analyzed using the Ultreon 2.0 software (Westford, MA, USA) to assess signs of vulnerability, luminal stenosis percentage, lesion length, proximal lesion diameter, and distal lesion diameter. Vulnerable plaques (VP) were defined as thin-cap fibroatheroma with a cap thickness of 0.075 mm or less, plaque rupture (PR), or plaque erosion. OCT imaging was performed after stent placement, and stent optimization was conducted based on the findings of whether malapposition was observed. On average, OCT imaging prolonged the intervention by 20 min. Figure 1 exemplifies OCT imaging with plaque erosion, plaque with a 0.05 mm fibrous cap thickness and plaque rupture.

### 2.7. The Decision for Revascularization

For the FFR group, revascularization decisions were based solely on FFR measurements. If the FFR was >0.8, the procedure was concluded, and the patient received maximal medical treatment. If the FFR was ≤0.8, a stent was placed.

For the FFR + OCT group, if the FFR was >0.8, the revascularization decision was based on OCT findings. If there were no vulnerable plaques, the procedure was concluded, and the patient received maximal medical treatment. If OCT imaging indicated VP, then the patient underwent revascularization. If the FFR was ≤0.8, the patient underwent revascularization regardless of OCT findings.

### 2.8. Postrevascularization Procedures

After the procedure ended, the arterial sheath was removed, and hemostasis was performed either with a hemostasis device for the radial artery or manual hemostasis for the brachial and femoral arteries, completed with a compression bandage. The patient’s mean hospitalization days were between 6 and 10 days. The patient was properly hydrated to prevent contrast media renal toxicity. Standard treatment for acute coronary syndrome was administered, as well as personalized treatment depending on each patient’s cholesterol, LDL, HDL, and triglyceride levels. 

### 2.9. Statistical Analysis

GraphPad Prism 9.0 software (GraphPad Software Inc., San Diego, CA, USA) was used for statistical analysis. Prior to statistical analysis, all data were checked for normality. The results were expressed as numbers and percentages. Statistical significance, expressed as *p*, was set at 0.05.

### 2.10. Ethics

This study was carried out in accordance with the code of Ethics of the World Medical Association (Declaration of Helsinki). All patients gave written informed consent, and the study protocol was approved by the ethics committee of the hospital.

## 3. Results

The clinical baseline characteristics of the study population are listed in Table 1.

**Figure 2:**
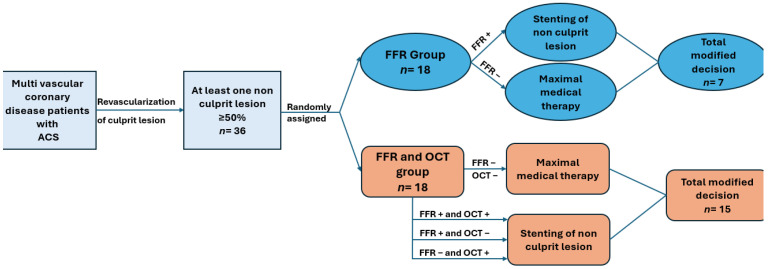
Flow chart exemplifying the study design. The study population consisted of patients admitted to the cardiology clinic with acute coronary syndrome who, following coronary angiography, were found to have multivessel coronary artery disease with at least one stenosis of 50% or more in a different vessel from the one responsible for the ACS. Patients were randomly assigned to two groups. In the FFR group, revascularization of non-culprit lesions was based on FFR analysis, while in the FFR and OCT groups, revascularization of non-culprit lesions was based on both FFR analysis and vulnerability features identified by OCT. In total, FFR analysis led to a change in the therapeutic decision in 7 cases in the FFR group, whereas FFR and OCT analysis led to a change in the therapeutic decision in 15 cases in the FFR and OCT groups.

The flow chart exemplifying the study design is shown in Figure 2.

### 3.1. Plaque Characteristics of Non-Culprit Lesions

The number of non-culprit lesions was comparable between the two groups, with 23 lesions in the FFR group and 30 in the FFR and OCT groups. Statistical analysis revealed no significant differences between the groups in terms of lesion location, balloon predilatation, location of predilatation, and a total number of stent placements (Table 2). However, in the FFR group, only one case required stent optimization, whereas in the FFR and OCT groups, OCT imaging indicated that 8 (44.44%) cases needed stent optimization: two in the right coronary artery, three in the anterior descending artery, and two in the circumflex artery. This difference was statistically significant (*p* = 0.0178; Relative Risk = 0.1765; 95% CI: 0.03113 to 0.7305).

### 3.2. Figures, Tables, and Schemes

There was no statistically significant difference between the two groups in the decision to stent based on FFR alone, with both groups having four cases each. Additionally, there was no statistical significance between the groups when analyzing the decision not to place a stent based on FFR findings. However, when considering OCT imaging, the medical decision was altered in 11 cases where FFR measurements were above 0.8 but the lesions were characterized as VP. Analyzing the total change in the decision to stent, 4 cases in the FFR group and 15 cases in the FFR and OCT groups (4 based on FFR and 11 on OCT) revealed a statistically significant difference (*p* = 0.0006; Relative Risk = 0.2556; 95% CI: 0.1013 to 0.5603). When analyzing the change in the total decision both to stent and not to stent, we observed a statistically significant difference, with Group 1 having 7 cases and Group 2 having 15 cases (*p* = 0.0153; Relative Risk = 0.4050; 95% CI: 0.2004 to 0.7698) (Table 3).

### 3.3. Per-Vessel Analysis 

The distribution and characteristics of lesions in the 11 cases where OCT imaging led to a modified clinical decision. The lesions were located at 4 in the right coronary artery, 5 in the left anterior descending artery, and 4 in the circumflex artery. The vulnerability criteria revealed that thin-cap fibroatheroma was present in 11 of the lesions. Plaque rupture and plaque erosion were each observed in four lesions (Table 4).

The fractional flow reserve measurements taken after adenosine administration were recorded for each artery. The mean FFR value for the RCA was 0.88 with a standard deviation of 0.07; for the ADA, it was 0.85 with an SD of 0.04; and for the ACX, it was 0.89 with an SD of 0.08.

**Figure 3:**
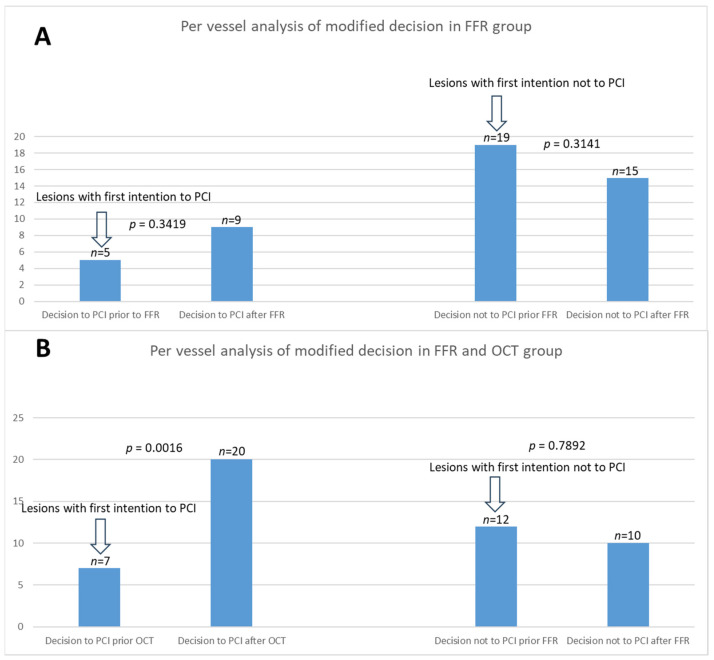
Per-vessel analysis of the impact of intracoronary imaging on modified therapeutic decisions. (**A**) (left) Increase in revascularization decisions from 5 to 9 following FFR in lesions with first intention to PCI. (right) Decrease in revascularization decisions from 19 to 15 after performing FFR in lesions with the first intention not to PCI. (**B**) (left) Increase in revascularization decisions from 7 to 20 following OCT in lesions with first intention to PCI. (right) Decrease in revascularization decisions from 12 to 10 after performing FFR in lesions with the first intention not to PCI.

**Figure 4:**
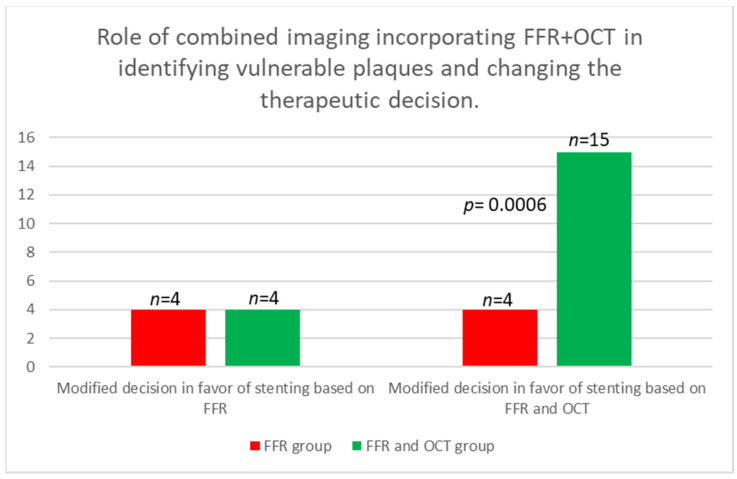
Modified decision in favor of stenting based only on FFR in both groups (**left**). Modified decision in favor of stenting based on FFR and OCT (**right**).

**Figure 5:**
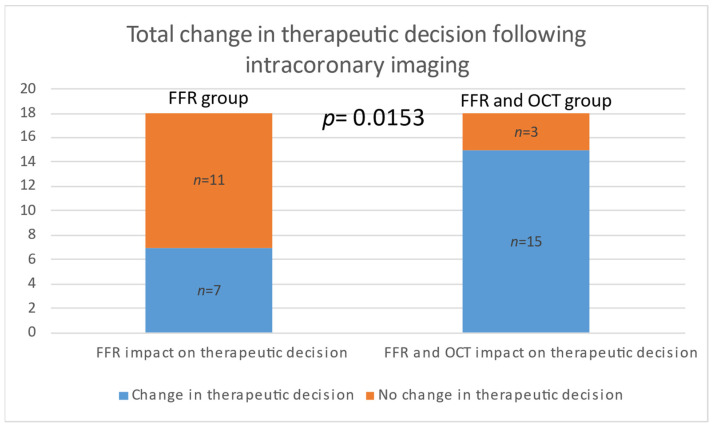
Total change in the therapeutic decision following intracoronary imaging. Change in treatment strategy in 7 out of 18 cases in the FFR group (**left**) and in 15 out of 18 cases in the FFR and OCT groups (**right**).

The per-vessel distribution of the analysis of the modified decision in both groups is illustrated in Figure 3. The figure shows a comparative analysis of the comprehensive change in treatment strategies based on FFR and OCT across both groups. Figure 4 summarizes the shift in decision to stent when incorporating OCT imaging. Figure 5 summarizes the overall shift in therapeutic decisions across both groups.

## 4. Discussion

Our study aimed to evaluate the impact of optical coherence tomography on decision-making in patients undergoing coronary revascularization based on fractional flow reserve assessments. The results provide insights into the clinical and procedural implications of incorporating OCT alongside FFR in the management of non-culprit lesions in acute coronary syndrome.

### 4.1. Integration of OCT in Clinical Decision-Making and Its Impact on Patient Outcomes

This is the first study to demonstrate that a combined imaging approach, including OCT and FFR, may increase the precision of coronary revascularization decisions and may identify dangerous plaques that remain undetected by isolated FFR assessment alone.

This study identified an important difference between the two groups in terms of stent optimization. In the FFR group, only one case required stent optimization, whereas OCT imaging indicated that eight cases required such optimization in the FFR and OCT groups, distributed across various coronary arteries, and this difference was statistically significant (*p* = 0.0178).

Fractional flow reserve has been extensively validated in numerous clinical studies as a dependable index for guiding decisions on coronary revascularization in the catheterization laboratory. Several pivotal studies have underscored the reliability of FFR in clinical settings, demonstrating its effectiveness and safety in determining whether to defer or proceed with coronary interventions based on the severity of ischemia [6,7,8,9].

Numerous studies have validated optical coherence tomography for its efficacy in detecting and characterizing vulnerable plaques, particularly thin-cap fibroatheromas, which are associated with heightened cardiovascular risk [10,11,12,13]. 

The integration of OCT significantly influenced clinical decision-making in our study. While there was no significant difference between the groups in the decision to stent based solely on FFR values, OCT imaging led to modifications in medical decisions in 11 cases where FFR values were above 0.8, but lesions were characterized as vulnerable plaques. This shift resulted in a higher number of cases requiring stents when OCT was added to FFR, as evidenced by the significant difference in total decision changes (*p* = 0.0006). Furthermore, when considering the decision change in both directions (from no stent to stent and from stent to no stent), significant differences were observed between the groups, underscoring OCT’s impact on treatment strategies (*p* = 0.0153).

### 4.2. Importance of Plaque Vulnerability Assessment

Our outcomes were consistent with findings by Jan-Quinten Mol et al., who investigated patients presenting with acute coronary syndrome and FFR-negative non-culprit lesions. Their research underscored that the presence of high-risk plaques in such lesions significantly correlates with poorer clinical outcomes, particularly an increased incidence of unplanned revascularizations [14]. This correlation highlights the critical importance of plaque vulnerability assessment in clinical decision-making. Our study supports the notion that integrating optical coherence tomography alongside fractional flow reserve measurements provides valuable insights into lesion characteristics beyond ischemic severity alone. By identifying high-risk features such as thin-cap fibroatheromas, plaque rupture, or erosion, OCT enables more precise risk stratification and guides optimal therapeutic strategies. Furthermore, our findings emphasize the potential of OCT to influence clinical management decisions by identifying lesions that may benefit from more aggressive therapeutic approaches. This approach aims to reduce adverse clinical events and enhance overall patient outcomes in the context of coronary artery disease. 

Furthermore, our study corroborates the findings of the LightLab Initiative, which demonstrated that OCT workflow influenced lesion assessment and PCI decision-making in 86% of cases, primarily affecting pre-PCI lesion evaluation and treatment strategy planning, leading to a more aggressive approach. Post-PCI OCT modified stent optimization decisions in 31% of cases, compared to our study, where stent optimization was performed in 44.44% of cases. This underscores the value of OCT-based decision-making in identifying high-risk plaques. Integrating OCT into routine clinical practice offers significant benefits in optimizing PCI strategies and ultimately improving patient care in the management of coronary artery disease [15].

### 4.3. Study Limitations

This study has several limitations. First, there is a potential for over-stenting due to the use of OCT in guiding PCI. OCT, while providing detailed insights into plaque morphology and lesion characteristics, may lead to more aggressive treatment decisions, such as additional stent placements, based on findings that may not necessarily translate into clinical benefit over the long term. Secondly, there are no long-term follow-up data in our study. Without extended monitoring, we cannot assess the sustained effectiveness or potential complications associated with OCT-guided interventions. This absence of follow-up limits our ability to evaluate the true impact of OCT on patient outcomes beyond the immediate post-procedural period.

## 5. Conclusions

Our findings demonstrate that OCT, when used in conjunction with FFR, enhances the precision of coronary revascularization decisions by identifying and characterizing vulnerable plaques that may not be detected by functional assessments alone. This approach represents a paradigm shift in coronary artery disease risk stratification, potentially leading to improved patient outcomes through targeted interventions. Future studies should further explore the long-term clinical benefits and cost-effectiveness of integrating OCT into routine clinical practice.

These insights contribute to the evolving landscape of interventional cardiology, where precise lesion characterization plays an increasingly pivotal role in optimizing therapeutic strategies for ACS patients. 

## Figures and Tables

**Table 1:** Baseline characteristics of the study population.

	Group 1 (FFR) (*N* = 18)	Group 2 (FFR&OCT) (*N* = 18)	*p*-Value
Age, mean ± SD	60.70 ± 10.71	59.76 ± 9.89	ns
Sex, male, *n* (%)	17 (94.44%)	14 (77.77%)	ns
Diabetes mellitus n (%)	4 (22.22%)	5 (27.77%)	ns
Smoker * *n* (%)	7 (38%)	4 (22.22%)	ns
Hypercholesterolemia *n* (%)	10 (55.55%)	6 (33.33%)	ns
Hypertension *n* (%)	14 (77.77%)	12 (66.66%)	ns
Family history of heart disease *n* (%)	6 (33.33%)	5 (27.77%)	ns
History of ACS *n* (%)	3 (16.66%)	2 (11.11%)	ns
Prior PCI *n* (%)	2 (11.11%)	2 (11.11%)	ns
Type of ACS			
STEMI *n* (%)	6 (33.33%)	11 (61.11%)	ns
N-STEMI *n* (%)	8 (44.44%)	2 (11.11%)	ns
UA *n* (%)	4 (22.22%)	5 (27.77%)	ns

ACS—acute coronary syndrome; PCI—percutaneous coronary intervention; STEMI—ST elevation myocardial infarction; N-STEMI—non-ST elevation myocardial infarction; UA—unstable angina; *—past or present; ns—non-significant.

**Table 2:** Non-culprit lesion analysis.

Number of Non-Culprit Lesions	Group 1 (FFR) *N* = 23	Group 2 (FFR&OCT) *N* = 30	*p* Value
Location			
RCA *n* (%)	9 (39.12%)	8 (26.66%)	ns
LAD *n* (%)	9 (39.12%)	12 (40%)	ns
LCX *n* (%)	5 (21.73%)	10 (33.33%)	ns
Total number of balloon predilatation	5 (21.73%)	5 (16.66%)	ns
RCA *n* (%)	3 (13.04%)	0 (00.00%)	ns
ADA *n* (%)	1 (4.34%)	2 (6.66%)	ns
ACX *n* (%)	1 (4.34%)	3 (10%)	ns
Total number of stent placement	11 (47.82%)	22 (73.33%)	ns
RCA *n* (%)	4 (17.39%)	5 (16.66%)	ns
ADA *n* (%)	5 (21.73%)	10 (33.33%)	ns
ACX *n* (%)	2 (8.69%)	7 (23.33%)	ns

RCA—right coronary artery; LAD—anterior descending coronary artery; LCX—circumflex artery; ns—non-significant.

**Table 3:** Modified decision based on OCT and FFR.

Modified Decision	Group 1 (FFR) *N* = 18	Group 2 (FFR&OCT) *N* = 18	*p* Value
To stent based on FFR *n* (%)	4 (22.22%)	4 (22.22%)	ns
To stent based on OCT *n* (%)		11 (66.66%)	
Total modified decision to stent	4 (22.22%)	15 (88.88%)	0.0006
Not to stent based on FFR *n* (%)	3 (16.66%)	0 (00.00%)	ns
Total modified decisions *n* (%)	7 (38.88%)	15 (88.88%)	0.0153

FFR—fractional flow reserved; OCT—optical coherence tomography; ns—non-significant.

**Table 4:** Per-vessel analysis in patients with modified decisions based on OCT.

Location	*N* = 13
RCA *n*%	4 (30.76%)
ADA *n*%	5 (38.46%)
ACX *n*%	4 (30.76)
Vulnerability criteria	
TCFA	11
Plaque rupture	4
Plaque erosion	4
FFR values	
RCA, mean ± SD	0.88 ± 0.07
ADA, mean ± SD	0.85 ± 0.04
ACX, mean ± SD	0.89 ± 0.08

TCFA—thin-cap fibroatheroma; RCA—right coronary artery; LAD—anterior descending coronary artery; LCX—circumflex artery.

## Data Availability

The data is not publicly accessible due to privacy concerns but may be provided upon request.

## List of Symbols

ACS	Acute coronary syndrome
CABG	Coronary artery bypass grafting
CAD	Coronary artery disease
ECG	Electrocardiogram
FFR	Fractional flow reserve
IVUS	Intravascular ultrasound
LAD	Anterior descending coronary artery
LCX	Circumflex artery
NS	Non-significant
MVD	Multivessel disease
NSTEMI	Non-ST-segment elevation myocardial infarction
OCT	Optical coherence tomography
Pa	Aortic pressure
PCI	Percutaneous coronary intervention
Pd	Pressure distal to a coronary stenosis
Pd/Pa	Ratio of the pressure distal to coronary stenosis to the aortic pressure
PR	Plaque rupture
RCA	Right coronary artery
RFR	Resting Full-cycle Ratio
STEMI	ST-segment elevation myocardial infarction
TCFA	Thin-cap fibroatheroma
UA	Unstable angina
VP	Vulnerable plaques
